# Atrial Fibrillation in the Young: A Neurologist's Nightmare

**DOI:** 10.1155/2015/374352

**Published:** 2015-04-02

**Authors:** Nikhil Aggarwal, Subothini Selvendran, Claire E. Raphael, Vassilios Vassiliou

**Affiliations:** ^1^Department of Cardiology, Royal Brompton Hospital, Sydney Street, London SW3 6NP, UK; ^2^Imperial College, London, UK

## Abstract

Atrial fibrillation (AF) is the most common sustained cardiac arrhythmia seen in clinical practice with prevalence in excess of 33 million worldwide. Although often asymptomatic and until recently considered a “benign” arrhythmia, it is now appreciated that thromboembolism resulting from AF results in significant morbidity and mortality predominantly due to stroke. Although an arrhythmia more commonly affecting the elderly, AF can also occur in the young. This review focuses on the impact of AF in the younger population and discusses the dilemmas of managing younger patients with AF.

## 1. Introduction

What do American former professional basketball player Larry Bird, former British Prime Minister Tony Blair, and Mother Theresa have in common? Surprisingly they have all suffered from atrial fibrillation (AF) [1–3]. AF is the most common sustained cardiac arrhythmia in clinical practice and affects 1-2% of the general population and in excess of 33 million worldwide [4]. Although AF becomes increasingly more prevalent with aging, it also affects young people; 0.5% of people under 40 years of age suffer from AF, with >5% of those over 65 and >10% of those more than 80 years of age being affected [5]. Despite AF being a documented cause of stroke for a long time [6], until recently it was still considered a relatively “benign” arrhythmia [7]. AF is a significant contributor to cardiovascular morbidity and mortality and is frequently more symptomatic and troublesome in the young, often causing significant impairment to quality of life [5, 8, 9]. But what does it mean when a person suffers from AF?

## 2. What Is AF?

In normal sinus rhythm there is a coordinated contraction of both atria and ventricles, but AF occurs when the atria of the heart receive random electrical impulses causing rapid and ineffective atrial contraction followed by irregularly irregular ventricular contractions [8, 10].

There are three types of AF: paroxysmal, persistent, and permanent. Paroxysmal AF can usually last from a few seconds up to a few days but the key feature is that this subsides spontaneously [11]. Persistent AF can last for any period of time; it is possible to restore sinus rhythm; however, an intervention is required either in the form of pharmacotherapy or electrical cardioversion or ablation for this to be achieved. Finally, permanent AF is present all the time and it is either not possible to restore sinus rhythm or deemed inappropriate to attempt cardioversion [11].

AF in the young can often be precipitated by hypertension, hyperthyroidism, or valvular heart disease as well as by certain lifestyle factors such as alcohol consumption and even smoking [12]. Avoiding these factors can prevent future AF attacks [5]. For the purposes of this review article the term young is used to capture adults under the age of 50. AF can cause many hemodynamic changes that can potentially lead to thromboembolic events, with stroke being the most dreaded complication. While stroke is more common in elderly patients with AF, it can also affect the younger population. In fact, one study by Sanak et al. shows the incidence of AF in a population of patients aged less than 50 with acute stroke to be as high as 11% with 70% of these having paroxysmal AF [13]. Strokes relating to AF can be more debilitating than non-AF related strokes [14], and this can be particularly life-changing, especially in the young. Therefore, not only does AF lead to mortality but it can lead to significant morbidity with increased distress suffered by the patient and the family and associated with an increased cost for the community [4]. It is also important to highlight that younger patients with AF have higher mortality than matched controls without a diagnosis of AF. Andersson and colleagues found patients who were admitted into hospital with incidental AF had worse prognosis when compared to patients without AF and that the risk of all-cause mortality was greater in the younger age group compared to the over-75-year-old population [15].

Despite its common prevalence, the management of AF can be difficult and controversial for the population as a whole. In recent years, however, by understanding the underlying pathophysiological mechanisms of this arrhythmia and the associated risks, various therapeutic options are currently utilized [8]. Nonetheless trying to balance the Scylla of thromboembolic stroke against the Charybdis of major bleeding complications in the young can be even more tricky, especially as young patients often have a lower thromboembolic risk and hence the absolute benefit of anticoagulation is less, whilst at the same time they are asked to commit to many years of anticoagulation with increasing overall absolute degree of bleeding risk.

## 3. Mechanism

For many years there were only speculative theories for the precise mechanism of AF. Over the last thirty years, however, many animal models and patient clinical studies have begun to clarify the underlying pathophysiology of this complex arrhythmia [16].

In normal sinus rhythm, islets of atrial myocardium and vascular smooth muscle in the coronary sinus and atrioventricular valves maintain synchronized electrical activity. In AF, ectopic foci occurring in “pockets” of atrial tissue within the pulmonary veins or atria can develop delayed depolarization leading to the arrhythmia's initiation. Many factors including sympathetic or parasympathetic stimulation, bradycardia, atrial premature beats, or tachycardia and acute atrial stretch [17] are thought to cause AF, although these factors are usually required in association with other contributors.

One study by Haïssaguerre and colleagues demonstrated impulses from a single focus in the pulmonary veins propagating to the atria as fibrillatory waves [18]. Furthermore, additional work from the same group supported this theory as they showed that radiofrequency ablation of the foci terminated AF [19]. However, newer evidence supports that structural as well as electrophysiological abnormalities lead to the development of AF. Frustaci and colleagues showed that atrial hypertrophy and fibrosis were more frequently present in patients with AF, supporting the theory that fibrosis itself could drive AF [20]. Furthermore, and more pertinent to young patients with AF, there is some recent evidence to suggest that, in patients with lone AF, the arrhythmia can be actually associated with structural atrial disease recently defined as “fibrotic atrial cardiomyopathy” [21]. In this situation, scarring within the atria may disrupt the normal pathways of conduction, leading to the initiation of atrial fibrillation. Imaging using cardiovascular magnetic resonance may allow detection of focal areas of fibrosis, both in the atria and in the ventricle. Similarly, areas of inflammation within the atria may also act as a focus for arrhythmia, producing “irritable” myocardium that is more arrhythmogenic. Unlike ventricular inflammation, imaging of atrial inflammation is challenging and this process may therefore be underappreciated. Another study by Stiles and colleagues confirmed atrial conduction abnormalities such as longer effective refractory period, longer conduction time along linear catheters, and slower conduction velocity as the cause of AF initiation and progression [22].

Athletes have also been shown to have higher prevalence of AF. It is thought that prolonged and strenuous exercise can predispose to AF [23] by relating to structural heart changes and in particular bigger left atria and left ventricles when compared to controls [24]. However, there could be an additional adrenergic and vagal component in this [25]. Furthermore, other structural heart conditions including hypertrophic cardiomyopathy (HCM) have been shown to have an increased incidence of AF. Darby and DiMarco, for example, have shown that the incidence of AF in HCM patients is in the region of 22% [26]. Older patients often present with hemodynamic instability due to the increasing prevalence of heart failure with age [27]; however, young patients with HCM and AF are at increased risk of symptomatic congestive heart failure, stroke, and even sudden cardiac death [26].

It is also well established that atrial electrophysiological properties are influenced by any changes in autonomic tone. Patients with structural heart disease often have AF, which is triggered by the sympathetic nervous system, for example, during episodes of sepsis or drug provocation, for example, with dobutamine. However those without structural heart disease can have AF mediated through the parasympathetic nervous system, through the vagus nerve. The exact mechanism by which foci are triggered is not known but it is thought that the close interaction between nerves and atrial myocytes can play a part [28]. In young patients particularly, it was found that there was a shift towards vagal dominance in lone AF and nocturnal paroxysmal AF [29]. Furthermore, it is known that in patients with paroxysmal AF and syncope there is an abnormal neural response even in sinus rhythm and AF can actually trigger vasovagal syncope [30].

Furthermore, inflammatory markers such as C-reactive protein and interleukin-6 have been implicated in AF initiation and progression. While an association has been identified, further work is required to confirm this and elucidate the underlying mechanisms [5, 31]. These findings together support the notion that AF is not merely a problem with the “electrics” of the heart but that structural and anatomical abnormalities predispose to AF development. Initiation of AF in an individual anatomically predisposed to AF may result from tachy- or bradyarrhythmias, infection, and inflammation.

Finally, perhaps a less emphasized cause of atrial fibrillation is due to specific genetic defects. The last decade in particular has seen an expansion in our understanding of the inherited form of the arrhythmia [32]. The familial form of AF is uncommon and research into its pathophysiology is still being carried out; however, it is more prevalent in younger patients [32, 33]. It is believed that a small percentage of cases are associated with changes in the* KCNE2*,* KCNJ2*, and* KCNQ1* genes, which are inherited through an autosomal dominant pattern [34]. These genes are responsible for ion transportation and play a critical role in maintaining the heart's normal sinus rhythm [34] and in the future they can perhaps aid in identifying young individuals at risk of developing AF at an earlier stage.

## 4. Diagnosing AF

The diagnosis of AF is suspected when the pulse is irregularly irregular and indeed in the United Kingdom all family practitioners are advised to check the pulse for any irregularity in everyone over the age of 65 [35]. An absent *α* wave in the jugular venous pulse is diagnostic of AF but sensitivity is poor. The diagnosis is confirmed by electrocardiography (EKG). The absence of P waves and an irregularly irregular ventricular response are diagnostic of AF on a 12-lead EKG. If the EKG demonstrates atrial flutter (when multiple P waves exist with each QRS complex), rather than AF, this should be treated in the same way as AF, as atrial flutter too can lead to thromboembolism. Although this review focuses on young patients with AF, the management of patients with atrial flutter is exactly the same [14]. In cases of suspicion of paroxysmal atrial fibrillation (or flutter) depending on the frequency of the palpitations, a 24-hour monitor or prolonged (5–7day) recorder or even an implantable loop recorder (ILR) can be used to identify AF [14]. Clinically, patients often present with breathlessness, palpitations, chest pain, and dizziness. Young patients are much more likely to present with palpitations than the elderly. However, the elderly are much more likely to present with anginal chest pain in contrast to young patients who are known to present with atypical chest pain [5]. Once AF has been confirmed the clinical examination should look for signs that may suggest predisposing factors such as hypertension, exophthalmos or goiter for thyrotoxicosis, heart murmurs for valvular heart disease, displaced apex beat for cardiomyopathy, prominent second heart sound (P2) supporting pulmonary arterial hypertension, respiratory wheeze, crackles or cyanosis supporting chronic obstructive pulmonary disease, and evidence for prior thromboembolic disease as a result of the AF.

## 5. How Does AF Lead to Stroke?

**Figure 1:**
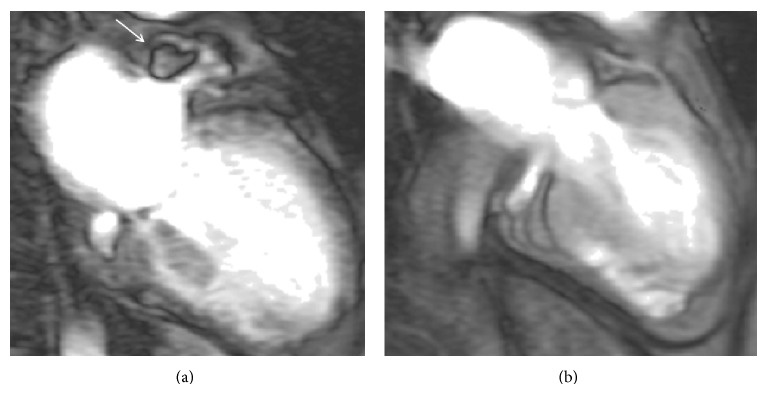
Cardiovascular magnetic resonance (CMR) image of a patient with AF in the early phase following gadolinium administration. On the left image demonstrates thrombus (white arrow) in the LAA and on the right 6 months later, following anticoagulation therapy with warfarin and confirming resolution of the thrombus.

The ineffective atrial contraction often leads to blood pooling in the atria. This pooling can then lead to thrombus formation due to blood stasis and subsequent fragmentation and dispersion in the arterial circulation can cause thromboembolic strokes and transient ischemic attacks (TIA) [4]. The most likely location for a thrombus is the left atrium and particularly the left atrial appendage (LAA). In fact for up to 90% of patients with nonvalvular AF in whom a thrombus has been identified, it is in the LAA as shown in Figure 1 [36]. The LAA is prone to blood stasis due to its long and tubular structure [4] and, furthermore, fibrillation can lead to decreased left atrial appendage flow velocities and decreased contractility [37], further increasing the risk of thrombosis in accordance to Virchow's triad [38].

Due to their large size, cardiac emboli flow to the intracranial vessels and in most cases can cause massive, superficial, single large striatocapsular or multiple infarcts in the middle cerebral artery territory. This stops the flow of oxygen to the brain and this brain anoxia leads to brain cell death [39]. In the posterior circulation, cardioembolism can produce Wallenberg's syndrome, cerebellar infarcts, top-of-the-basilar syndrome, multilevel infarcts, or posterior-cerebral-artery infarct [39].

It is however important to note that AF is not the only cause of cerebral infarction in the young. Although rare, a paradoxical embolism through a defect in the interatrial septum (such as a patent foramen ovale (PFO) or atrial septal defect (ASD)) can allow small emboli to pass from the venous to the arterial and then cerebral circulation causing a stroke [40, 41]. A PFO is commonly found in the healthy population with up to 25–30% of people having a PFO [42]. Therefore in any patient with stroke and presence of PFO this should not be automatically considered as causative and a search for other explanations for the stroke, including paroxysmal AF, should be initiated. Other rare causes for stroke in the young include vasculitis, Takayasu's arteritis, and Behçet's syndrome which can both cause thrombi [43], infections such as bacterial endocarditis [44], and myeloproliferative disorders particularly polycythemia rubra vera [45]. Patients may also have procoagulative states such as antithrombin III deficiency, protein C and S deficiencies, or hereditary conditions such as hyperhomocystinemia and factor V Leiden [46]. The use of oral contraceptive pills, or recreational drugs [47], cerebral hemorrhage secondary to congenital aneurysms, arteriovenous malformations, or hypertension are other recognized causes of stroke in the young [48].

## 6. How Does Stroke Affect Patients?

Following a stroke, patients can experience loss of vision, dizziness, trouble communicating, loss of balance, and difficulty swallowing [49]. Furthermore, they often describe a “lack of control” over their bodies, not knowing when or where the weakness will stop [50], as well as shock and fear when their bodies became weaker. This can lead to sleepless nights, full of anxiety, which is further aggravated by the fact that they may not be able to turn and move themselves [51]. Healthcare workers commonly come across stroke patients who have depression and this is even more frequent in the young [52]. Questions like “why bother to teach me how to dress? I won't be able to do it anyway” are often asked indicating a vulnerable population that could benefit from significant support [53]. Furthermore, perhaps more so in the young, patients often worry how their relationship with their partner, including sexual activity [54], and friends might change and whether people might start viewing them differently; as for those still in full-time education they are worried about how the stroke will affect their performance and consequently their future careers.

## 7. Preventing a Stroke in a Patient with AF

In every patient with AF, stroke is the most dreaded complication, and as such doctors and patients should work together to try and minimize the risk for this. It is not possible to eliminate the risk completely, but it is now possible to estimate relatively accurately the thromboembolic risk for each patient and therefore try and personalize medical therapy. Using a validated risk assessment score such as the CHA_2_DS_2_-VASc demonstrated in Table 1, which has been recently developed to help healthcare practitioners assess thromboembolic risk, clinicians can estimate this risk and have more accurate discussions with the patients [55]. They can assess the need of oral anticoagulants (OAC) and also other medical therapies to ensure other risk factors that could increase stroke risk (such as hypertension, hypercholesterolemia, and diabetes) are adequately controlled [14]. The HAS-BLED (hypertension, abnormal renal/liver function, stroke, bleeding history or predisposition, labile international normalized ratio, the elderly (> 65 years), and drugs/alcohol concomitantly) scoring system helps assess the individual's bleeding risk in AF patients to further support clinical decision-making regarding anticoagulants [56]. A score of greater than 3 shows the patient is very prone to bleeding and therefore must be monitored frequently [57]. After full assessment, family doctors, cardiologists, and increasingly neurologists are called to make a decision based on the thromboembolic risk of the individual and their bleeding tendency as to whether the benefits of anticoagulation are likely to outweigh the risks associated with it. It may well be that in a young patient with no evidence of any structural heart disease or hypertension anticoagulation is not started due to a lower risk score using traditional scoring systems for thromboembolic risk (e.g., CHA_2_DS_2_-VASc = 0 or 1) [58]. It is however important to consider anticoagulation in all patients, as OACs have been shown to have a major preventative role in cardioembolism in AF [59]. Since there is less evidence base in the young, we favor discussion between cardiology and neurology for individual cases. Although the risk score may give a lower annual risk of further stroke, it is important to appreciate that, in the younger person with a greater life expectancy, they are exposed to this risk for a longer period of time and it may therefore be appropriate to have a lower threshold for anticoagulation providing that the hemorrhagic risk is acceptable. Vitamin K antagonists are very effective in reducing stroke risk. In fact, studies have shown that dose-adjusted warfarin with a target INR of between 2 and 3 leads to a 64% decrease in the relative risk reduction of ischemic stroke [60]. Although warfarin is very effective, it has many interactions with other drugs and also many dietary interactions. Coupling this with the need for frequent blood monitoring, patients often find it very unappealing especially in the young who face anticoagulation for many years [61]. More recently, other novel oral anticoagulant (NOAC) drugs have been approved by Food and Drugs Administration (FDA) and European Medicines Agency (EMA) including dabigatran etexilate, a direct thrombin inhibitor, and apixaban and rivaroxaban, two direct factor Xa inhibitors [62]. These drugs have been shown to be at least as affective as warfarin with a possible lower risk of bleeding in older populations but have not been formally assessed in the younger patients who were perhaps underrepresented in these studies [63, 64]. Although extremely appealing, mainly because of the lack of requirement of blood monitoring, these drugs are fairly new and so all the possible side effects may not as yet be known [7]. This can be especially important in a young person with AF facing the prospect of anticoagulation for many decades and the possibility of rare idiosyncratic potentially serious side effects. Despite this, their current uptake has been increasing mainly due to their convenience and is supported by the current guidance [14, 65] but the long-term safety of NOACs in young people is still awaited. Antiplatelet medication such as aspirin and clopidogrel, which were once frequently used in the thromboembolic risk reduction of patients with AF, should only be used infrequently nowadays. The stroke risk reduction with a single antiplatelet agent is only modest at best (10% in a meta-analysis) [66] and as such these drugs can only be recommended for patients with minimal thromboembolic risk [14]. Naturally all reversible causes of AF and ischemic stroke in the young should be excluded before anticoagulation is started or continued indefinitely. Furthermore, young patients in particular should have regular risk assessments as the indication for anticoagulation may change. It is becoming more clear that with increasing OAC options available for AF this is becoming more complex. The decision to choose which anticoagulant for which patient assuming that the patient would benefit from anticoagulation is particularly difficult in the young. It is therefore important to discuss the more complex cases at a multidisciplinary setting including neurologists and cardiologists.

Historically, a rhythm control strategy (i.e., aiming to restore sinus rhythm) was considered superior to simply controlling the rate of ventricular escape rhythm in AF [67]. However, the AFFIRM trial showed that rate control was at least as good as rhythm control in symptomatic AF [68]. Controlling symptoms in young patients particularly with paroxysmal AF and rapid ventricular rates is difficult with just rate control however [67]. If patients continue to experience symptoms after at least one antiarrhythmic drug then consideration of AF ablation is recommended. Typically the ideal candidates for AF ablation are younger patients without structural heart disease and before persistent AF develops [67]. Catheter ablation has been found to be associated with shorter hospitalization, fewer complications, and favorable clinical outcomes in young patients with paroxysmal AF [69]. The cornerstone of radiofrequency ablation for AF is electrical pulmonary vein isolation, which isolates triggers of AF activity in the pulmonary vein and therefore stops AF. More recently increasingly complex ablation techniques have been used in an attempt to increase the success of the procedure including atrial substrate modification, targeting complex fractionated electrograms, ligament of Marshall ablation, ablation of ganglion plexi, linear ablation, focal impulse or rotor modulation, and rapid drivers/dominant frequency [70].

## 8. Investigations in Acute Stroke

In an acute stroke brain imaging with a computer tomography (CT) or magnetic resonance imaging (MRI) should be carried out immediately [71]. This can help exclude a hemorrhagic stroke and may identify an area of ischemia supporting the use of thrombolysis [72]. Other investigations, which can be helpful in identifying the cause of a stroke in a young person include: transthoracic echocardiography (TTE), prolonged EKG recording and CT or MRI angiography of the great vessels [73]. Transesophageal echocardiography (TEE) is indicated in patients where TTE is not diagnostic. A TTE can show dilated left atrium, right to left shunting, and possibly a PFO/ASD [74] but cannot however show a thrombus in the LAA, the leading cause of stroke due to AF which can be clearly seen with a TEE, cardiac MRI, or CT [14].

If all the above tests prove inconclusive and the stroke remains cryptogenic (cerebral ischemia/infarction due to unknown origin [73]), management becomes more complex as lack of knowledge of the cause of the stroke makes treatment more challenging.

## 9. Prevention of AF

Although a positive family history has been shown in up to a quarter of patients with AF, there are still many risk factors, which can be optimized to reduce the burden of AF [75–77]. Risk factors particularly pertinent to young patients include exercise, obesity, smoking, hypertension, and alcohol [5]. Long-term consumption of alcohol and binge drinking, which is particularly common in young people, has also been shown to increase the risk of AF by 37% and has been associated with as much as 60% of new onset AF in patients under the age of 65 [78, 79]. This is in contrast to older people where the major risk factors of AF include hypertension, coronary heart disease, heart failure, valvular heart disease, thyroid disorders, and diabetes [80]. Some of these risk factors can be reduced by maintaining a healthy lifestyle with regular mild-moderate physical activity and a balanced diet low in saturated fats and cholesterol. Blood pressure should be managed and a healthy weight maintained. Other underlying conditions including sleep apnea, thyroid disease, and diabetes, which can contribute to AF, should also be controlled [5]. The thromboembolic risk in AF in the young is more difficult to manage as the benefits of long-term anticoagulation should be balanced against the bleeding risk, as illustrated through the following cases.

*Case  1*. A 23-year-old male right-handed bricklayer with no significant past medical history presents to the emergency room (ER) on a Saturday night. He had been out drinking for the evening and he noticed some palpitations about 3 hours previously. On examination there was no hemodynamic instability and cardiac auscultation was normal. The pulse was irregularly irregular and the initial EKG showed AF with a heart rate of 130 bpm. A repeat EKG 30 minutes later showed spontaneous reversion to normal sinus rhythm. He was observed overnight where he remained in sinus rhythm and was discharged the following morning. He was referred to the joint cardiology/neurology AF assessment clinic in the hospital as well as being given lifestyle advice on binge drinking.

It is not an uncommon scenario to see patients presenting following significant binge drinking with AF. In this example, on taking a full history, the patient had in excess of 20 units of alcohol the evening prior to his admission in casualty. This reverted back to sinus rhythm without needing any pharmacotherapy. A 24-hour EKG monitoring prior to the clinic assessment showed normal sinus rhythm with no episodes of arrhythmias and his transthoracic echocardiogram was completely normal. In particular there was no valvular abnormality, his ventricular systolic function was normal, and his left atrial size was normal indicating that he was unlikely to have had significant continuous episodes of atrial fibrillation. His blood parameters including renal, liver, and thyroid functions were normal. There was no family history of cardiac disease or AF.

What needs to be addressed in the outpatient clinic is why he got AF and how he should be managed in the longer term, in terms of both arrhythmia prevention and management of his thromboembolic risk. In the absence of any structural heart abnormalities and biochemical abnormalities and with no family history it would be acceptable to consider the heavy alcohol intake on the day of the presentation to ER as the trigger of AF. Anticoagulation would not be recommended as the cause for his AF is reversible. He should refrain from drinking heavily and this is likely to prevent further AF attacks. This should be explained to him when seen in the outpatient appointment. At the same time his thromboembolic risk profile needs to be considered and if he has any evidence of hypertension or hypercholesterolemia, this should be treated accordingly. He should also be advised to present in ER in the future, ideally within 12 hours from the onset of any similar episodes (to facilitate chemical/electrical cardioversion if necessary), and importantly refrain from driving himself to ER in case of very rapid AF that could cause hemodynamic compromise. His family practitioner should be informed of this episode and involved in his future care, particularly in ensuring that there are no further episodes of fibrillation and that this person refrains from drinking excessively in the future. 

*Case  2*. A 24-year-old female right-handed semiprofessional tennis player presented with an acute attack of right facial and arm weakness. She consulted her family physician within an hour of the event. The family physician undertook an EKG and identified new onset AF. Her past medical history included well treated hypertension and migraines. The family physician was worried that this could represent an acute stroke and referred her to the neurology department for further investigations and management. 

Her risk factors for AF include hypertension and also significant exercise training [81]. The concern here in the acute phase is whether her weakness might represent migraine or a thromboembolic event. In this case an urgent brain MRI confirmed an acute ischemic stroke in the left middle cerebral artery distribution. However, although she had some weakness, this rapidly improved after initial presentation and did not meet criteria for acute stroke thrombolysis. She was admitted for observation and also had a TTE. The TTE demonstrated slightly dilated right ventricular dimensions with a dilated left atrium and normal left ventricular systolic function, in keeping with an “athlete's heart.” There was no evidence of an intra-atrial shunt at rest or on bubble study. Given the AF and recent stroke, anticoagulation with warfarin as first line would be appropriate. To reduce the risk of hemorrhagic transformation of the cerebral infarction, anticoagulation is usually started 14 days after the event [14] and only once blood pressure is controlled. To reduce thromboembolic risk, anticoagulation should continue lifelong as her thromboembolic risk is high (CHA_2_DS_2_-VASc = 4, conferring a risk of adjusted stroke risk of 4% per year without anticoagulation as shown in Table 1). In terms of restoring sinus rhythm it may be appropriate to refer her for electrophysiological studies and percutaneous pulmonary vein isolation/AF ablation. Although this could restore sinus rhythm and prevent potential cardiomyopathy relating to the AF, she will need to remain on anticoagulation lifelong as the risk of thromboembolism will remain even if an AF ablation is apparently successful [14]. She should also continue to have outpatients review (ideally once annually) to assess her thromboembolic risk and ensure that any additional factors (e.g., hypertension) are well controlled.

*Case  3*. A 20-year-old male left-handed engineering student presents with signs of an acute right middle cerebral artery stroke. An urgent computer tomography (CT) of the brain excluded brain hemorrhage and he was promptly thrombolysed in ER. The following morning when assessed he was found to have made a good recovery. His EKG demonstrated AF and a TTE showed normal left ventricular systolic function with normal size left atrium and additionally demonstrated a patent foramen ovale (PFO). His management was discussed in a joint neurology/cardiology meeting. 

This is a complex case of a young man presenting with a stroke and two possible etiologies: the AF and PFO. It would be important to identify which one is responsible for the stroke as it might facilitate different treatment. If we hypothesize that the PFO was responsible for the stroke then this would have meant that the venous thrombus (usually from the lower limbs) moved from the right (venous) side of the heart through the atrial septum to the left (arterial) side of the heart and embolized in the brain. It would be useful to undertake bilateral lower limb ultrasonography to rule out a deep vein thrombosis. If he had any symptoms to suggest a pulmonary embolus then a CT pulmonary angiogram could be undertaken to look for this. If a source for a peripheral thrombus can be identified then it would be logical to assume that the stroke was secondary to embolization through the PFO and support percutaneous closure of this to prevent further similar events [82]. A TEE could also be undertaken; this will allow further visualization of the left atrium and left atrial appendage looking for any evidence of thrombus there, which would suggest that the AF is the likely culprit for the stroke. This could also be used to further visualize the size and anatomy of the PFO. In the absence of peripheral thrombus and given that PFOs are very common, affecting up to 25–30% of the population [83], it would be reasonable to consider that the PFO is not related to this stroke, in the presence of AF, a more likely contributor. The AF should therefore be managed with anticoagulation 14 days after the event (as in the case above) and further management for restoration of sinus rhythm could be considered. Chemical or electrical cardioversion or electrical catheter ablation could be considered at a later stage. Like Case  2, this patient should be anticoagulated for life in view of his thromboembolic risk (CHA_2_DS_2_VASc = 2, conferring a risk of adjusted stroke risk of 2.2% per year without anticoagulation) and reviewed, ideally annually initially in the outpatient clinic, to ensure that other factors that could contribute to his thromboembolic risk are addressed.

## 10. Conclusion

AF is the most common cardiac arrhythmia contributing to significant morbidity and mortality. Although a disease predominantly of the elderly, AF also affects young people. Thromboembolic disease and particularly stroke in the young can lead to increased long-term morbidity, which can affect relationships, education, and employment. Anticoagulation can reduce the risk of thromboembolism in the young with AF; however such a decision must not be taken lightly in view of the long-term risk of bleeding seen with all anticoagulants. Close liaison and discussion between patients, family doctors, cardiologists, and neurologists can allow the best management of patients with AF and particularly in young ones.

## Figures and Tables

**Table 1:** Demonstrating the recently described CHA_2_DS_2_-VASc thromboembolic risk score for patients with AF. It has a maximum of nine points where more points equate to higher thromboembolic risk as shown in Table 2.

CHA_2_DS_2_-VASc	Condition	Points allocated
C	Congestive heart failure or left ventricular (LV) dysfunction with ejection fraction (EF) ≤40%	1

H	Hypertension	1

A_2_	Age ≥ 75	2

D	Diabetes	1

S_2_	Transient ischemic attack (TIA), stroke, or other thromboembolisms	2

V	Vascular disease (e.g., peripheral artery disease, myocardial infarction, and aortic plaque)	1

A	Age 65–74	1

Sc	Sex category, that is, female	1

**Table 2:** Using the CHA_2_DS_2_-VASc score, an annual adjusted risk score for stroke can be estimated and the benefits of anticoagulation considered.

CHA_2_DS_2_-VASc score	Risk (annual adjusted risk)
0	0%
1	1.3%
2	2.2%
3	3.2%
4	4.0%
5	6.7%
6	9.8%
7	9.6%
8	6.7%
9	15.2%
